# Editorial for Special Issue: “Liposomal and Lipid-Based Drug Delivery Systems and Vaccines”

**DOI:** 10.3390/pharmaceutics16020238

**Published:** 2024-02-06

**Authors:** Elena L. Vodovozova

**Affiliations:** Shemyakin–Ovchinnikov Institute of Bioorganic Chemistry, Russian Academy of Sciences, ul. Miklukho-Maklaya 16/10, 117997 Moscow, Russia; elvod.ibch@yandex.ru

Liposomes and lipid-based supramolecular systems have been used in clinical practice for more than 30 years as drug carriers and vaccines for the treatment of oncological diseases and infections. Starting with liposomal formulations for small-molecule drugs (amphotericin B, doxorubicin, daunorubicin, etc.), lipid nanoparticles with siRNA used for gene therapy (Onnpatro^®^) and mRNA vaccines (from Pfizer/BioNTech and Moderna) have become available in recent years. Due to their clinical success, extensive research on lipid carriers is ongoing. The studies delve into the creation of new formulations to expand the range of active pharmaceutical ingredients for delivery, including targeting, the behavior of various formulations in the bloodstream and immune system reactions, as well as many other aspects related to the fabrication and use of lipid carriers.

The original research papers published in this Special Issue present new perspectives of the development of liposomal small-molecule drugs designed for different indications and modes of administration [1,2,3], anti-cancer immunogenic liposomes [4], lipid-based formulations with siRNA [5], and mRNA for effective transfection in vitro [6,7] and in vivo [8]. One review [9] considers the rational design of liposomal gene delivery systems that target the folic acid receptor.

A research team from the Arbuzov Institute of Organic and Physical Chemistry, Russian Academy of Sciences, Kazan, Russia, developed liposomes on the basis of egg phosphatidylcholine and imidazolium surfactants that can penetrate through the blood–brain barrier and deliver an encapsulated reactivator of acetylcholinesterase (AChE) pralidoxime chloride (2-PAM) [1]. After the intravenous administration of liposomal 2-PAM in rats poisoned with an organophosphorous inhibitor of AChE paraoxon (POX), AChE reactivation by 25% was observed, and in poisoned mice, the formulation caused a significant decrease in POX-induced neuronal death in the hippocampus.

Researchers from Lomonosov Moscow State University, in collaboration with colleagues from Sechenov University and the Semenov Federal Research Center for Chemical Physics, proposed a lipid–polymer system for the delivery of antibacterial and antifibrotic agents, administered through inhalation, for the treatment of long-term pulmonary diseases [2]. They designed fluoroquinolone and pirfenidone formulations based on anionic liposomes decorated with mucoadhesive mannosylated chitosan, studied the physicochemical patterns of drug interactions with gel-phase dipalmitoylphosphatidylcholine bilayers doped with cardiolipin and cholesterol using infrared spectroscopy (attenuated total reflectance method), and showed that the polymer shell stabilizes liposomes and decelerates the release of the drugs. After a single endotracheal administration in mice, the lipid–polymer formulation of moxifloxacin showed a prolonged accumulation of this drug in lung tissues compared with the controlled intravenous or endotracheal administration of a free drug.

Another study involving researchers from several Moscow institutions—the Shemyakin-Ovchinnikov Institute of Bioorganic Chemistry and the Institute of General Pathology and Pathophysiology of the Russian Academy of Sciences, N.F. Gamaleya Research Institute, and V.N. Orekhovich Institute of Biomedical Chemistry—demonstrates the effect of the protein corona of targeted antitumor liposomes on internalization by human endothelial cells under the conditions of modeling microvessels in a microfluidic device [3]. The ability of fluid-phase liposomes, loaded with the lipophilic prodrug of melphalan and decorated with a tetrasaccharide selectin ligand SiaLe^X^, to target activated endotheliocytes was maintained under microflow conditions. However, in the flow of human serum, the level of liposome uptake by cells decreased. Proteomic analysis of liposome–protein complexes revealed a correlation between the content of SiaLe^X^ and some apolipoproteins, likely including nonspecific competitors of selectins. Among them, apoC1, one of the most positively charged plasma proteins, was the most abundant in the corona of targeted liposomes. Thus, when systemically administering targeted liposomes or other drug delivery systems, the shielding effect of the protein corona should be taken into account.

A study by researchers from several U.S. institutions—University of Alaska Anchorage; Johns Hopkins University, Baltimore; Henry M. Jackson Foundation for the Advancement of Military Medicine, Silver Spring; and Mayo Clinic, Rochester—contributed to the development of a smart (and resource-saving) approach to creating targeted anticancer vaccines. This approach utilizes endogenous complement C3 to deliver antigens and Toll-like receptor (TLR) agonists directly to C3 receptors on the immune antigen presenting cells in a manner that mimics pathogenic uptake [4]. Pegylated gel-phase liposomes contain a lipid with a terminal orthopyridyl disulfide group, which readily forms a disulfide bond with the sulfhydryl group of complement C3 upon entering the bloodstream. In this study, a synthetic human mucin-1 (MUC1) 100 mer peptide and TLR7/8 agonist, co-encapsulated in C3-liposomes, provoked a significant antibody response in transgenic MUC1-tolerant mice, compared to non-encapsulated antigens. The co-encapsulation of TLR4, TLR7/8, and TLR9 agonists with MUC1 not only further increased antibody responses but also significantly increased T cell responses. This immunization strategy has the potential for antigen-specific cancer immunotherapy since MUC1 is overexpressed and hypo-glycosylated in a high percentage of carcinomas.

Researchers from the University of KwaZulu-Natal, Durban, South Africa, studied nanosized lipoplexes formulated from cationic liposomes composed of equimolar ratios of original cholesterol-based cytofectins and a neutral helper lipid, dioleoylphosphatidylethanolamine (DOPE), with and without a polyethylene glycol stabilizer, and siRNA to silence the HER2/neu oncogene [5]. One of the non-PEGylated siRNA lipoplexes induced the highest level of HER2/neu silencing at the mRNA and the protein levels (10,000-fold and >111.6-fold decreases, respectively), exceeding that of commercial Lipofectamine 3000 by more than a 4-fold reduction in mRNA expression in HER2/neu overexpressing breast adenocarcinoma SKBR-3 cells. Minimal dose-dependent cytotoxicity, biocompatibility, and excellent transfection efficiency make these liposomes a promising vehicle for siRNA in vivo.

Two articles concern the design of mRNA lipoplexes for effective transfection [6,7]. Researchers from St. Petersburg Polytechnic University and Smorodintsev Research Institute of Influenza, St. Petersburg, Russia, in collaboration with the Lomonosov Institute of Fine Chemical Technologies, Moscow, and the Global Virus Network, Baltimore, USA, examined the effect of the ratio between the original polycationic lipid, gemini-amphiphile (containing two cholesterol anchors and the central module of spermin), and the helper lipid DOPE on the delivery efficiency of two model mRNAs to hamster fibroblasts and cultured human adenocarcinoma cells [6]. They found that lipoplex-mediated transfection efficiency also depends on the lipid-to-mRNA ratio. A well-chosen ratio of all components of the complex lipoplex allowed for a more efficient delivery of both mRNA coding firefly luciferase and mRNA-eGFP into the cells, compared with commercial Lipofectamine MessengerMax. Liposomes based on the studied cationic gemini-lipid may be of interest for the future development of mRNA vaccines.

A research team from the Shemyakin-Ovchinnikov Institute of Bioorganic Chemistry and the Moscow Engineering Physics Institute tested a series of multicomponent lipid nanoparticles as a platform for the formation of lipoplexes for RNA transfection [7]. They investigated a cationic component, oleoylcholine; the well-known components, DOTAP (dioleoyltrimethylammonium propane) and DOPE; and a number of surfactants and natural oils, which make up the core of nonpolar lipids, in order to obtain long-term storage nanoparticles for on-demand complexation with RNA. However, oleoylcholine destabilized the secondary structure of mRNA, causing a decrease in transfection efficiency, and mRNA lipoplexes without a hydrophobic nucleus were more effective in transfecting HEK 293T cells of human embryonic kidney compared with nucleated particles. Interestingly, the functionalization of DOTAP-DOPE liposomes with GM3 ganglioside did not affect the transfection efficiency of HEK 293T cells and MDAMB-231 cells of human breast cancer, but led to a dramatic increase in the transfection of SW 620 cells of human rectal cancer, despite these cells being difficult to transfect, while the expression of the GM3 receptor gene (CD169) in all the studied cells was confirmed by PCR. These GM3-liposomes also effectively delivered siRNA against the GPR55 receptor, which is involved in tumor progression, to triple-negative cells MDAMB-231.

A study on mRNA-transfection presented by the researchers from Hoshi University, Tokyo, Japan, is one of the most advanced contributions to this Special Issue [8]. These researchers proposed a modified ethanol injection method to prepare mRNA lipoplexes, which are formed by simply mixing a lipid–ethanol solution with an mRNA solution. Six cationic lipids, including DOTAP, three neutral helper lipids (DOPE, dioleoylphosphatidylcholine, and cholesterol) and polyethylene glycol–cholesteryl ether, were used to generate 18 mRNA lipoplexes. Several of the latter lipoplexes showed a high protein expression in HeLa cells with firefly luciferase mRNA, and two of these, with relevant sizes of about 200 nm, were studied for in vivo transfection. For one formulation, the systemic injection in mice resulted in high protein expression in the lungs and spleen. Furthermore, this formulation with OVA mRNA induced an increase in OVA-specific IgG1 levels upon intravenous immunization. The ethanol content in the dosage for IV injection is only a quarter of LD50 (in mice), so this simple method of preparing mRNA lipoplexes without special equipment appears to be worth noting for use for in vivo mRNA transfection.

A review [9] by the researchers from the Lomonosov Institute of Fine Chemical Technologies discusses the targeting of lipid-based formulations with therapeutic nucleic acids through folate receptors overexpressed in many tumor cells. After a brief review of non-viral delivery systems with non-covalent folic acid binding, the following aspects are analyzed: problems with the rational design of folate-containing lipoconjugates, their optimal content within the delivery system, the physicochemical characteristics of lipoplexes, namely, the size and zeta-potential required for maximal delivery efficiency of nucleic acids into target cells. The review contains 93 references, approximately half of which are papers from the last five years, which indicates the ongoing interest and development of tumor targeting through lipoplexes conjugated with folic acid.

Overall, the reports presented in this Special Issue highlight the importance of further research on liposomes and lipid-based systems for drug delivery, which ultimately aim to improve the treatment and diagnosis of tumors and infectious diseases. As a Guest Editor, I am deeply grateful to all the authors who responded to the call to contribute to this Special Issue with the results from their most recent high-quality research and critically evaluated their manuscripts during the review process.
